# Supplementary material for: Allosteric cross-talk in chromatin can mediate drug-drug synergy
Source: Nat Commun. 2017 Mar 30;8:14860. doi: 10.1038/ncomms14860 (PMC5379107; doi:10.1038/ncomms14860)
Supplement: Supplementary Information — Supplementary Figures, Supplementary Tables and Supplementary References [file ncomms14860-s1.pdf]

Supplementary Table 1. Data collection and refinement statistics for NCP treated with RAPTA-T

	**RAPTA-T–NCP**
**Data collection***	
Space group	$P2_12_12_1$
Cell dimensions	
a (Å)	106.68
b (Å)	109.82
c (Å)	182.34
Resolution (Å)	2.81–60.8 (2.81–2.96)
R_{merge} (%)	5.9 (50.1)
$I / \sigma I$	15.5 (2.3)
Completeness (%)	96.2 (78.2)
Redundancy	6.6 (4.3)
**Refinement**	
Resolution (Å)	2.81–60.8
No. reflections	49,694
R_{work} / R_{free} (%)	20.8 / 24.1
No. atoms	12,077
Protein	6,086
DNA	5,939
Solvent	16
Adduct	36
B-factors (Å^2)	101
Protein	72
DNA	130
Solvent	120
Adduct	177
R.m.s. deviations	
Bond lengths (Å)	0.010
Bond angles (°)	1.50

Single crystal data, from a 20 hour-incubation with 0.25 mM RAPTA-T.
Values in parentheses are for the highest-resolution shell.

Supplementary Table 2. RAPTA-T and auranofin binding sites in crystals of the NCP

**Agent Treatment (mM, h)**			**Resolution (Å)**	**Anomalous Peak (σ)**			
RAPTA-T	AUF	Duration		RU1	RU2	AU1	AU1'
Data collected at λ=1.04 Å							
0	1	24	2.50				
0	1	68	2.50				
0	1	92	2.38				
0.5	2	17	2.38	3.0	3.5	15.8	17.8
0.5	2	43	2.60	2.3	3.0	13.4	14.5
0.5-1*	2	40	2.87	3.2	3.2	19.6	17.9
0.5-1*	2	40	2.88	3.6	4.7	24.5	25.3
Data collected at λ=1.50 Å							
0.5	2	16	2.50	3.8	3.7	14.6	13.7
0.5	2	43	2.75	3.3	3.2	7.5	8.4
0.5-1*	2	40	2.80	6.7	4.3	23.1	22.3

Peak heights are based on anomalous difference electron density maps
* 22 h incubation at 0.5 mM, followed by 18 h incubation at 1 mM

Supplementary Table 3. Data collection and refinement statistics for NCP treated with RAPTA-T and auranofin

	**RAPTA-T/AUF–NCP**
**Data collection***	
Space group	$P2_12_12_1$
Cell dimensions	
a (Å)	106.73
b (Å)	109.95
c (Å)	182.04
Resolution (Å)	2.80–48.9 (2.80–2.95)
R_{merge} (%)	3.7 (43.3)
$I / \sigma I$	23.2 (1.6)
Completeness (%)	95.8 (76.6)
Redundancy	5.0 (2.0)
**Refinement**	
Resolution (Å)	2.80–48.9
No. reflections	48,472
R_{work} / R_{free} (%)	21.5 / 23.6
No. atoms	12,093
Protein	6,086
DNA	5,939
Solvent	16
Adduct	52
B-factors (Å^2)	101
Protein	70
DNA	132
Solvent	121
Adduct	145
R.m.s. deviations	
Bond lengths (Å)	0.007
Bond angles (°)	1.46

Single crystal data, from a 40 hour-incubation with 2 mM Auranofin and 0.5-1.0 mM RAPTA-T (22 hours at 0.5 mM and 18 hours at 1.0 mM).
Values in parentheses are for the highest-resolution shell.

Supplementary Table 4. Statistical occurrence (expressed as % of the total simulation time) of close contacts between AUF and the histone protein residues during MD of the RAPTA-T/AUF–NCP system. Data are calculated during the last ~200 ns of MD, as well as over the entire length of the production runs (i.e., 600 ns), with the associated average and standard deviation. Hydrophobic contacts were counted for nonpolar atoms separated by at most 4.0 Å[1]. Data are reported for the front and rear of the NCP.

**RAPTA-T/AUF-NCP**								
	FRONT				REAR			
Histone H3'	Last 200 ns	Prod. run	Average	Stdev	Last 200 ns	Prod. run	Average	Stdev
A135	20.2	18.9	19.6	0.9	30.7	29.7	30.2	0.7
R134	60.4	63.4	61.9	2.1	73.9	74.5	74.2	0.4
E133	32.5	30.4	31.4	1.5	38.7	36.4	37.5	1.6
G132	7.7	6.7	7.2	0.7	9.3	10.3	9.8	0.7
R131	59.0	60.1	59.5	0.8	71.0	70.4	70.7	0.4
I130	60.5	59.7	60.1	0.6	60.0	55.6	57.8	3.1
R129	74.5	69.8	72.1	3.3	74.1	72.3	73.2	1.2
R128	73.1	76.6	74.9	2.5	73.2	70.3	71.7	2.0
A127	20.0	18.6	19.3	1.0	20.0	22.4	21.2	1.7
L126	84.0	86.4	85.2	1.7	83.5	84.6	84.1	0.7
E125	51.5	48.5	50.0	2.1	50.0	52.6	51.3	1.8
I124	60.5	62.2	61.4	1.2	57.2	58.8	58.0	1.1
D123	36.0	39.4	37.7	2.4	33.2	31.7	32.5	1.1
K122	85.3	85.3	85.3	0.0	80.2	79.6	79.9	0.4
Histone H3	last.200	prod.	average	stdev	last.200	prod.	average	stdev
A102	20.1	19.6	19.8	0.3	20.1	19.2	19.6	0.6
L103	60.0	62.3	61.1	1.6	60.0	62.4	61.2	1.7
F104	55.3	56.4	55.8	0.8	55.3	53.3	54.3	1.4
E105	38.0	36.7	37.4	0.9	38.0	36.6	37.3	1.0
D106	27.3	25.4	26.3	1.3	27.3	26.4	26.9	0.7
T107	36.7	34.6	35.7	1.5	36.7	32.6	34.7	2.9
N108	33.9	35.6	34.7	1.2	33.7	30.4	32.0	2.3
L109	74.3	72.3	73.3	1.4	74.1	76.5	75.3	1.7
C110	30.8	28.7	29.7	1.5	30.7	31.5	31.1	0.6
A111	23.0	21.4	22.2	1.2	23.0	22.5	22.8	0.4
I112	82.7	80.2	81.5	1.8	81.8	78.4	80.1	2.4
H113	Covalently bound							
A114	33.3	31.4	32.4	1.4	33.3	30.1	31.7	2.3
K115	69.1	65.3	67.2	2.7	69.0	68.7	68.8	0.2

Supplementary Table 5. Statistical occurrence (expressed as % of the total simulation time) of close contacts between AUF and the histone protein residues during MD of the AUF–NCP system. Data are calculated during the last ~200 ns of MD, as well as over the entire length of the production runs (i.e., 600 ns), with the associated average and standard deviation. Hydrophobic contacts were counted for nonpolar atoms separated by at most 4.0 Å[1]. Data are reported for the front and rear of the NCP.

**AUF-NCP**								
	FRONT				REAR			
Histone H3'	Last 200 ns	Prod. run	Average	Stdev	Last 200 ns	Prod. run	Average	Stdev
A135	34.4	32.1	33.2	1.6	30.0	32.5	31.3	1.8
R134	73.1	71.3	72.2	1.3	61.4	63.4	62.4	1.4
E133	38.3	40.2	39.3	1.3	32.2	28.6	30.4	2.6
G132	9.3	10.3	9.8	0.7	7.7	9.8	8.8	1.5
R131	71.9	75.2	73.5	2.3	59.0	56.7	57.9	1.6
I130	50.1	48.2	49.1	1.3	50.1	50.1	50.1	0.0
R129	62.3	59.2	60.7	2.2	61.8	61.8	61.8	0.0
R128	60.5	60.2	60.4	0.2	60.0	60.0	60.0	0.0
A127	16.6	12.5	14.5	2.9	16.6	16.6	16.6	0.0
L126	69.3	70.2	69.8	0.6	69.6	69.5	69.5	0.1
E125	42.8	41.9	42.4	0.7	43.9	42.4	43.2	1.1
I124	50.2	48.8	49.5	1.0	49.8	45.6	47.7	3.0
D123	29.7	32.4	31.1	1.9	29.7	23.4	26.6	4.5
K122	69.8	64.2	67.0	3.9	70.1	64.6	67.4	3.9
Histone H3	last.200	prod.	average	stdev	last.200	prod.	average	stdev
A102	16.6	17.8	17.2	0.8	16.6	18.6	17.6	1.4
L103	49.7	46.4	48.1	2.4	49.8	46.3	48.0	2.5
F104	46.1	42.9	44.5	2.3	46.0	48.9	47.5	2.0
E105	31.6	28.7	30.1	2.0	31.6	32.3	31.9	0.5
D106	22.6	23.3	23.0	0.5	22.6	22.3	22.5	0.2
T107	30.5	27.8	29.1	1.9	30.4	32.4	31.4	1.4
N108	28.0	28.9	28.4	0.6	28.0	23.4	25.7	3.2
L109	62.1	60.3	61.2	1.3	62.0	62.3	62.1	0.2
C110	25.5	23.2	24.3	1.6	25.4	23.4	24.4	1.4
A111	19.2	22.1	20.7	2.1	19.1	18.9	19.0	0.2
I112	68.4	64.3	66.4	2.9	67.8	69.7	68.7	1.3
H113	Covalently bound							
A114	27.6	25.8	26.7	1.3	27.7	29.7	28.7	1.4
K115	57.2	55.4	56.3	1.3	57.4	56.5	57.0	0.7

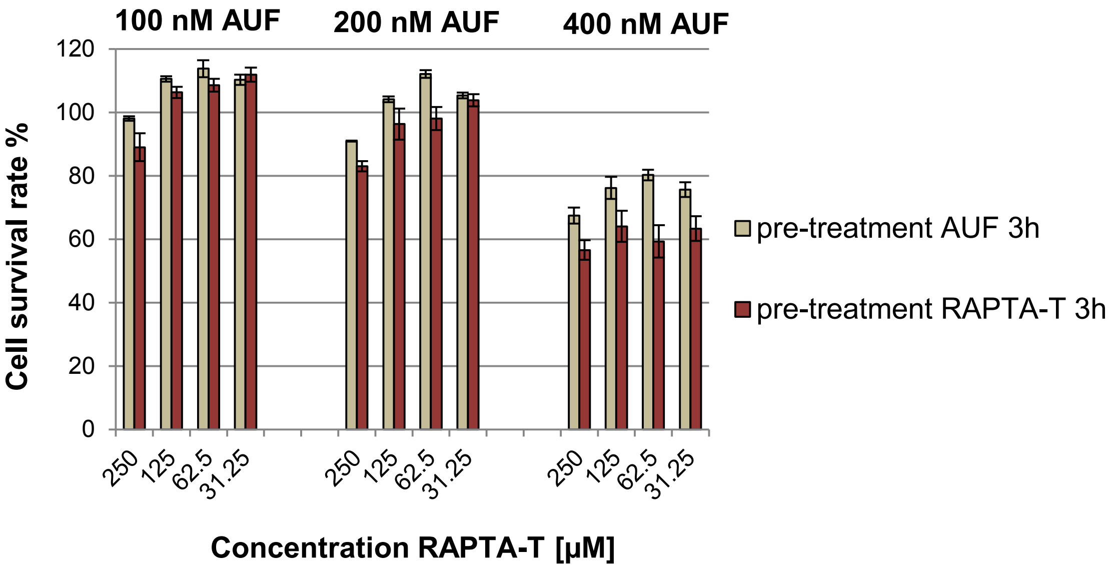

Supplementary Figure 1. Pre-treatment with RAPTA-T sensitizes ovarian cancer cells towards AUF. A2780 ovarian cancer cells were pre-incubated with different doses of either RAPTA-T or AUF for 3h before incubating the cells with the respective other drug for a further 48h (mean±s.d., *n*=3).

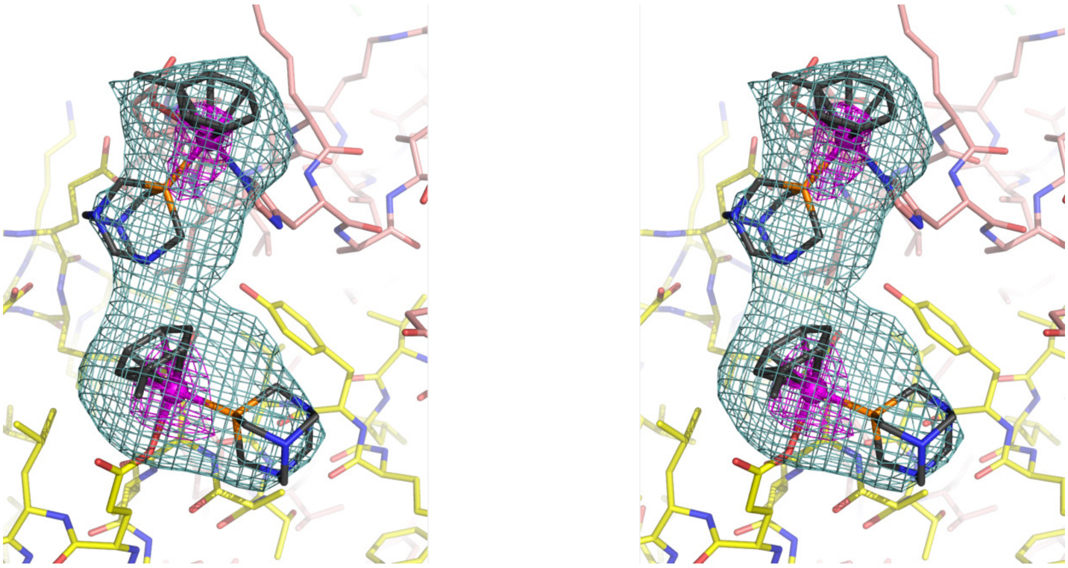

Supplementary Figure 2. Experimental electron density corresponding to the RAPTA-T adducts in the X-ray crystal structure of RAPTA-T–NCP, in stereo view. An $F_O{-}F_C$ omit electron density map (teal; contoured at 2.5σ; RAPTA-T atoms omitted from the model) and an anomalous difference electron density map (magenta; contoured at 4.5σ) are superimposed onto the refined model. RAPTA-T sites RU1 and RU2 are at bottom and top, respectively.

a

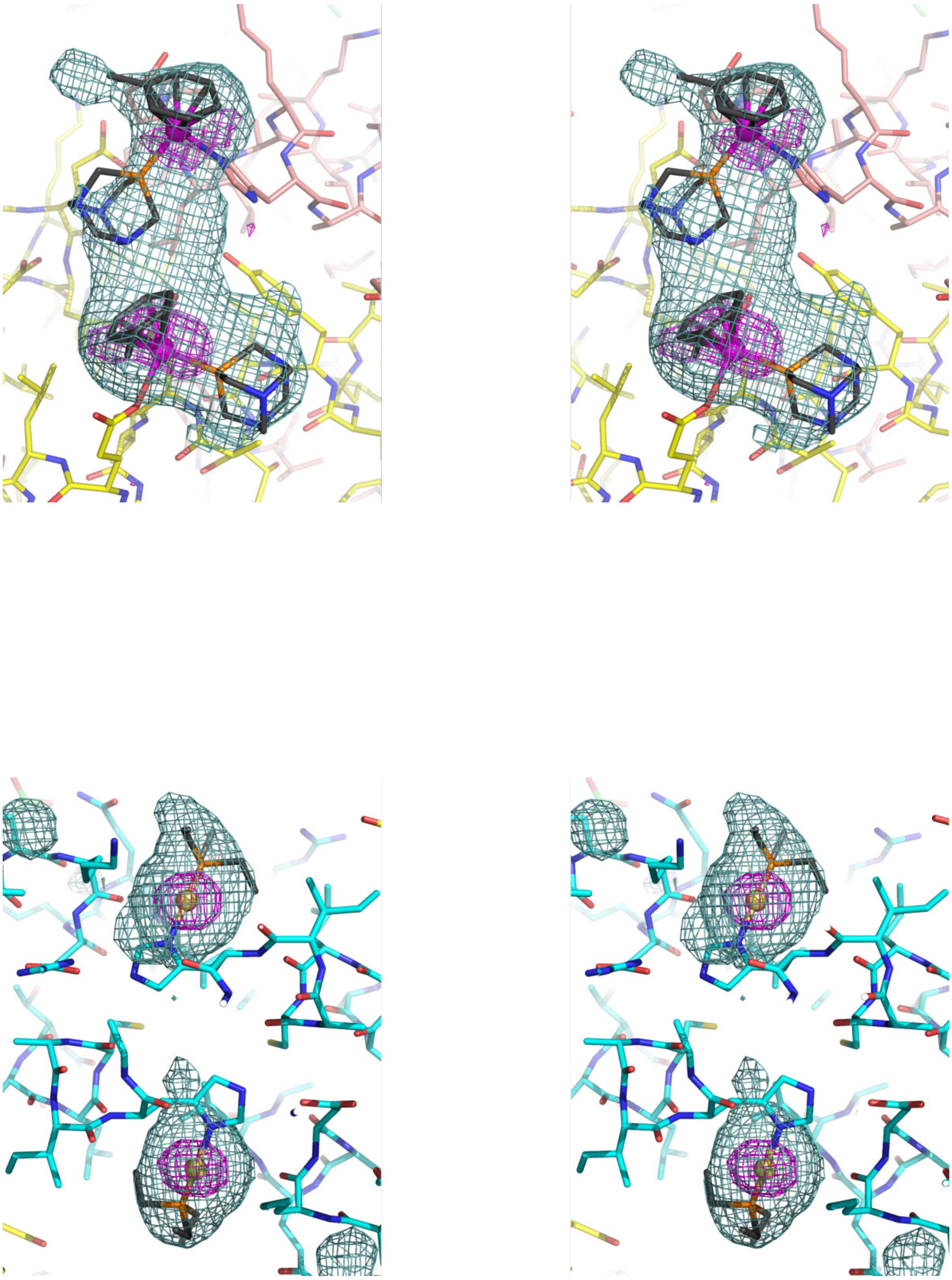

b

Supplementary Figure 3. Experimental electron density corresponding to the RAPTA-T and AUF adducts in the X-ray crystal structure of RAPTA-T/AUF–NCP, in

stereo view. (**a**,**b**) An F_O−F_C omit electron density map (teal; contoured at 2.5σ [a,b]; RAPTA-T and AUF atoms omitted from the model) and an anomalous difference electron density map (magenta; contoured at 3.2σ [a] and 11σ [b]) are superimposed onto the refined model. (**a**) RAPTA-T sites RU1 and RU2 are at bottom and top, respectively. (**b**) AUF sites AU1 and AU1' are at top and bottom, respectively.

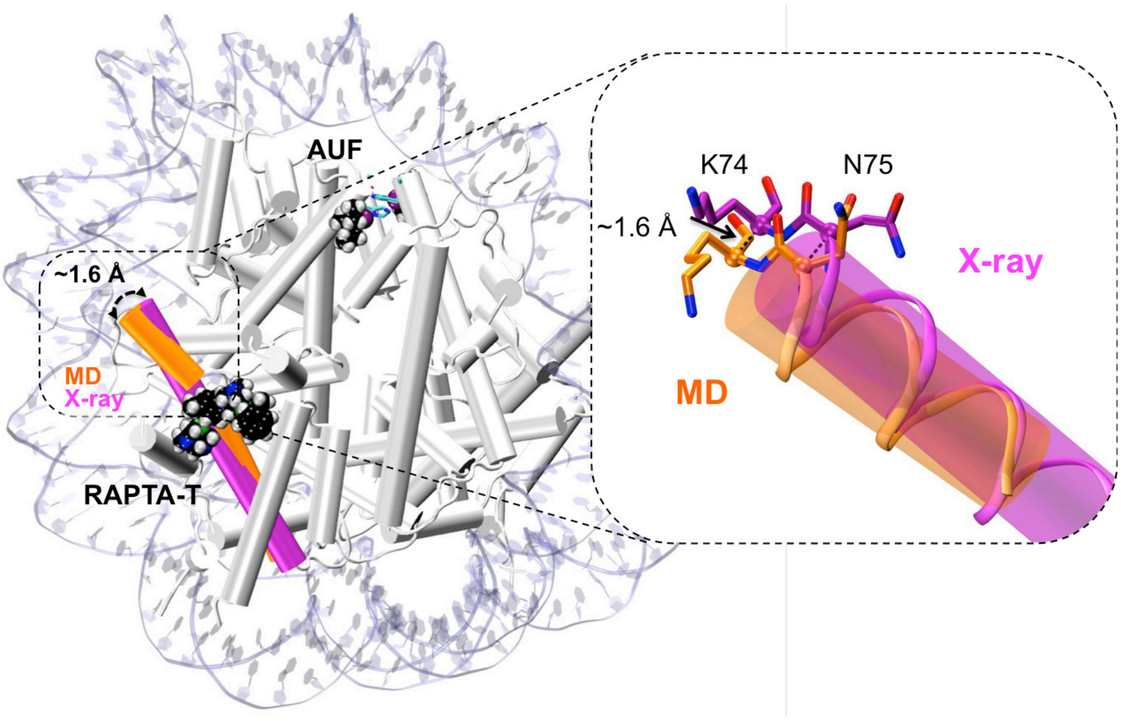

Supplementary Figure 4. X-ray structure of the RAPTA-T/AUF–NCP complex superposed with a selected MD frame of the system. The long α-helix of the H2A histone is shown in magenta (X-ray structure) and orange (MD frame), highlighting the kink induced by the presence of RAPTA-T adducts. This kinking occurs during the early stages of the MD simulation (i.e., during the equilibration phase) and results in a shift of ~1.6 Å of the end of the α-helix (calculated by measuring the distance between the Cα atoms of the K74 and N75 residues) of the H2A histone with respect to the X-ray structure. A close view is shown on the right.

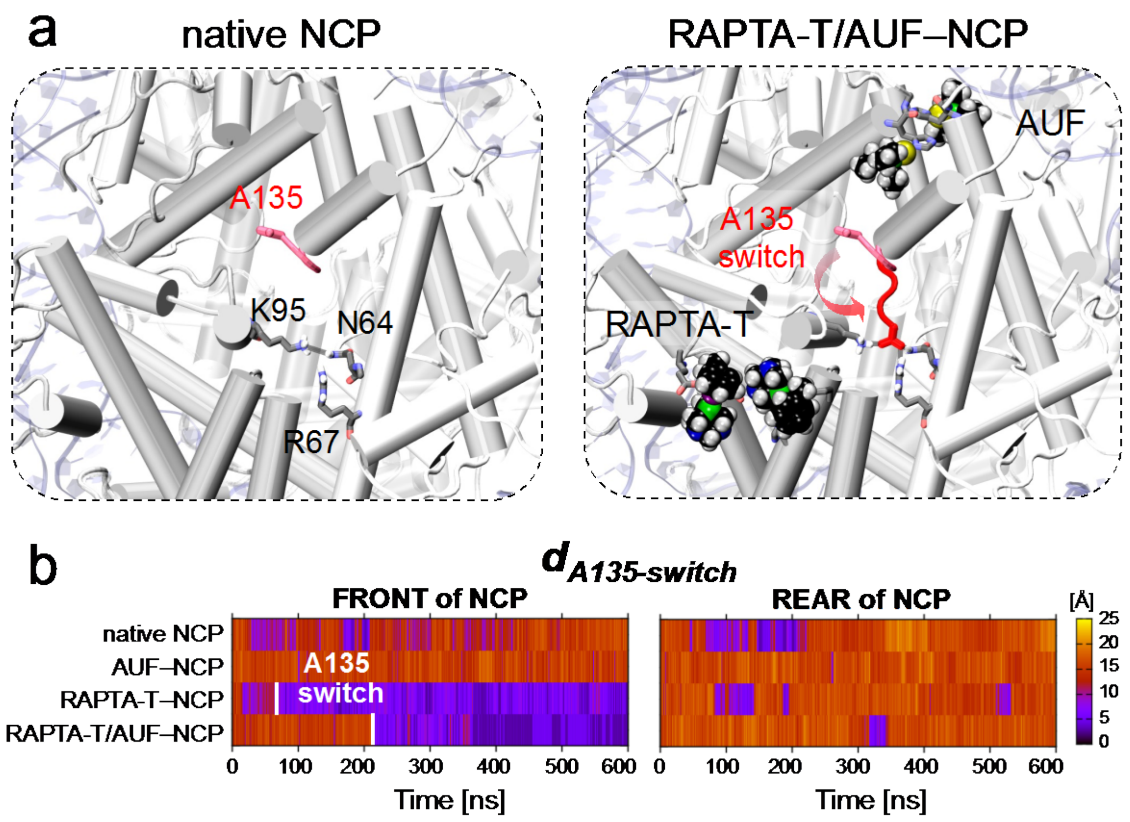

Supplementary Figure 5. **(a)** Snapshots from MD simulations of the NCP (left panel) and the RAPTA-T/AUF–NCP (right panel) systems, showing the conformational switch of the H3 C-terminal residue A135 occurring in the presence of RAPTA-T adducts (right). The protein histones are shown as cartoon, the DNA is represented as ribbons. RAPTA-T and AUF (black) are shown in a space-filling representation. In the right panel, an arrow indicates the conformational switch of A135, which is shown as sticks in both its initial (pink) and final (red) configurations. The H2A K95, H4 N64 and H4 R67 residues, which H-bond with A135 after its conformational switch are also shown. **(b)** Time evolution of the $d_{A135\text{-}switch}$ distance at the front (left) and rear (right) of the NCP, AUF–NCP, RAPTA-T–NCP and RAPTA-T/AUF–NCP systems, color-coded according to the scale on the right. The $d_{A135\text{-}switch}$ distance is defined as the distance between the Cα atom of H3 A135 and the center of mass (COM) of the H4 N64 and R67 residues.

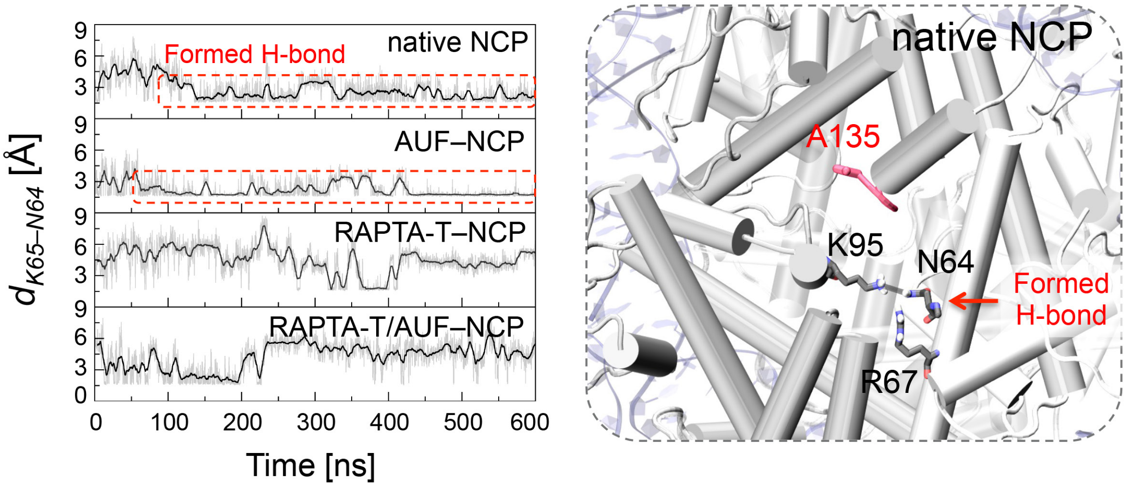

Supplementary Figure 6. Time evolution of the donor–acceptor H-bond distance between the H2A K95 and H4 N64 residues ($d_{K95–N64}$), reported for the NCP, AUF–NCP, RAPTA-T–NCP and RAPTA-T/AUF–NCP systems. A red box highlights the formed H-bond in the absence of RAPTA-T adducts (NCP and AUF–NCP). The box on the right shows the formed K95–N64 H-bond within the NCP. The protein histones are shown as cartoon, the DNA is represented as ribbons. H3 A135 (red) and the H2A K95, H4 N64 and H4 R67 (black) residues are shown as sticks.

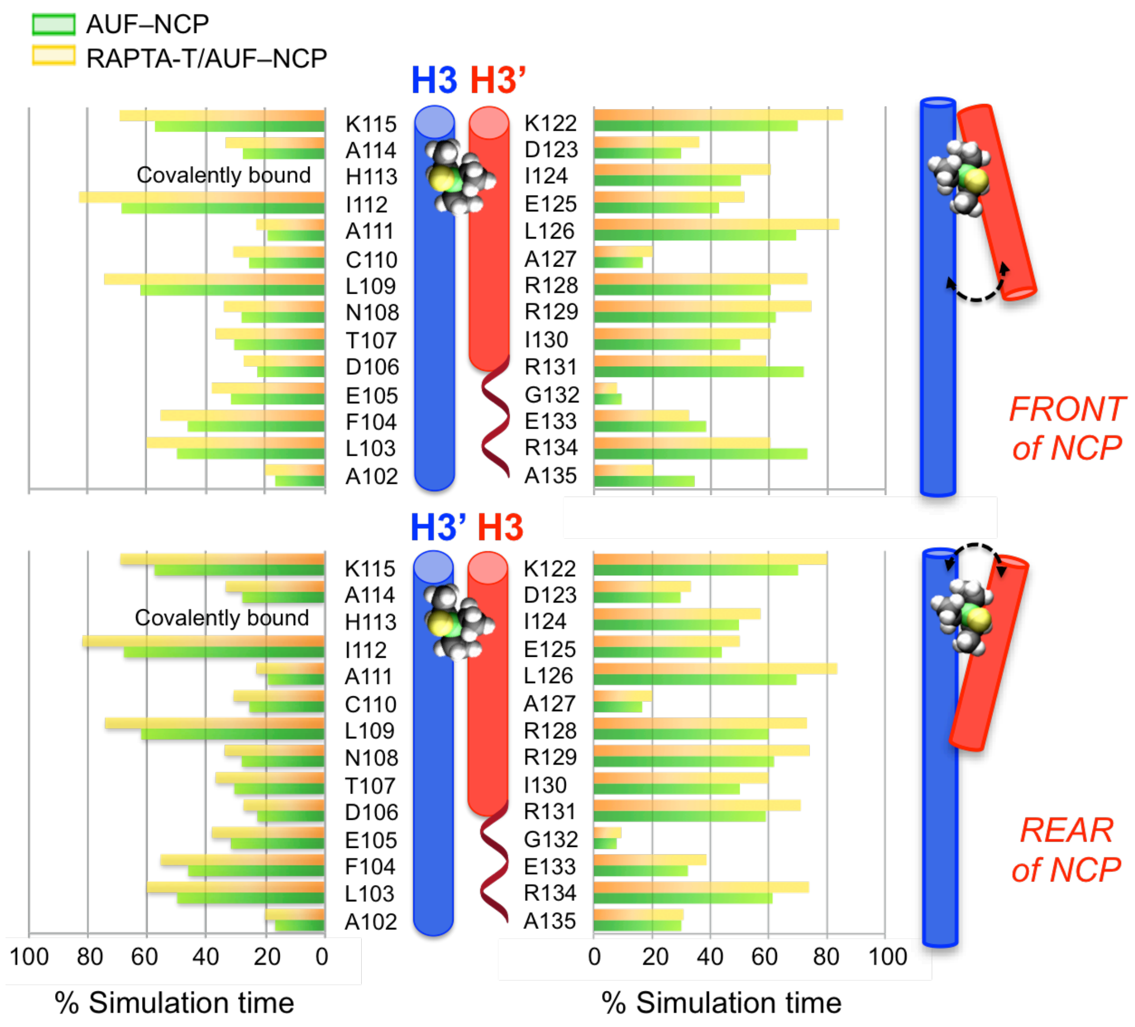

Supplementary Figure 7. Statistical occurrence (expressed as % of the total simulation time) of close contacts between AUF and the histone protein residues during the last ~200 ns of MD of the AUF–NCP (green bars) and RAPTA-T/AUF–NCP (yellow bars) systems. Hydrophobic contacts were counted for nonpolar atoms separated by at most 4.0 Å[1]. Data are reported for the front (top) and rear (bottom) of the systems. A schematic cartoon representation of the H3/H3’ interfaces, allocating AUF at the front and rear of the NCP structure, is also shown. The average difference in statistical occurrence for the close contacts between the two systems is 8.6 ± 1.4%. Full data including standard deviation is given in Supplementary Tables 4 and 5.

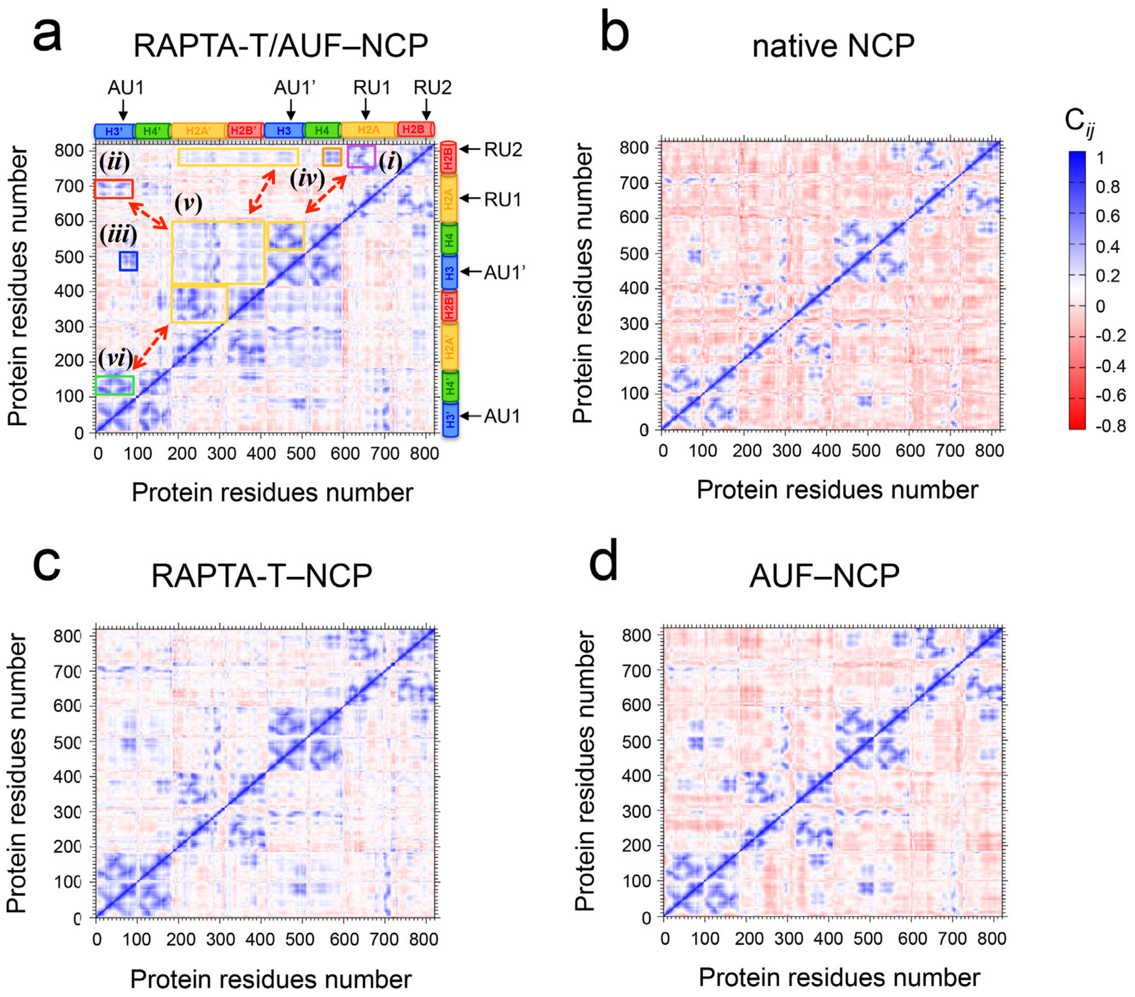

Supplementary Figure 8. Cross-correlation matrices of the fluctuations of the Cα atoms (C_{ij}) around their mean positions, calculated for the last ~200 ns of MD simulations of the RAPTA-T/AUF–NCP **(a)**, NCP **(b)**, RAPTA-T–NCP **(c)**, and AUF–NCP **(d)** systems. The extent of correlated ($0 > C_{ij} < 1$) and anticorrelated ($-1 > C_{ij} < 0$) motions is color-coded according to the scale on the right. On the C_{ij} plot of the RAPTA-T/AUF–NCP system **(a)**, RAPTA-T and AUF sites are indicated, as well as the H2A, H2B, H3 and H4 histones. Regions indicating highly correlated motions are highlighted with boxes (*i*–*vi*). Full details are reported in the main text.

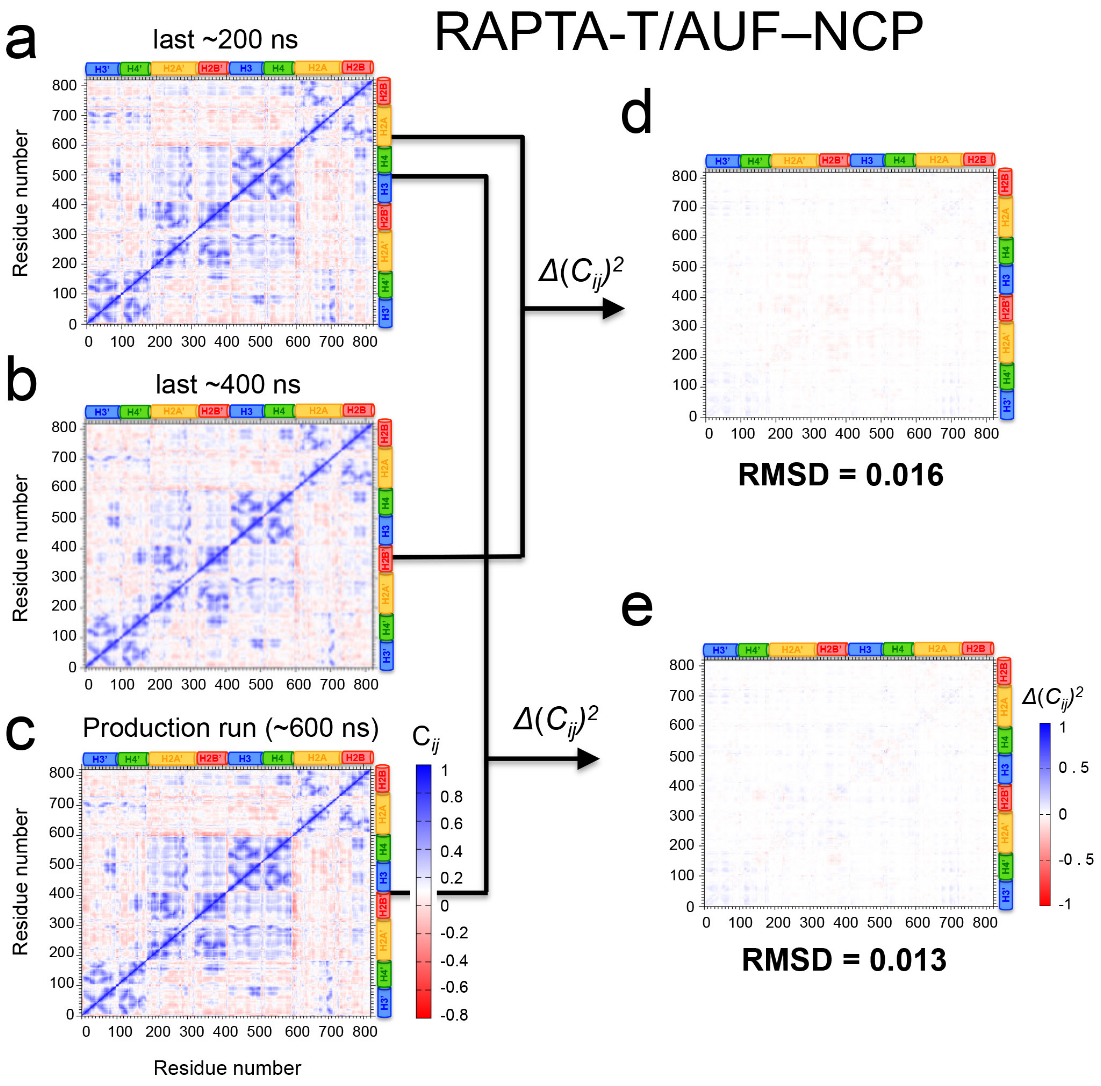

RMSD	native NCP	RAPTA-T–NCP	AUF–NCP	RAPTA-T/ AUF–NCP	One RAPTA-T
last 200ns – last 400 ns	0.018	0.015	0.012	0.016	0.017
last 200ns – 600 ns	0.015	0.017	0.015	0.013	0.018

Supplementary Figure 9. Cross-correlation matrices (C_{ij}) of the RAPTA-T/AUF–NCP system, calculated over three different time windows, considering the last ~200 ns of MD **(a)**, the last ~400 ns of MD **(b)**, as well as the entire production run, consisting of ~600 ns of MD **(c)**. The difference matrix $\Delta(C_{ij})^2$ between the square of the C_{ij} matrix elements in **(a)** and **(b)** is given in **(d)**, while the $\Delta(C_{ij})^2$ matrix between the square of the C_{ij} matrices in **(a)** and **(c)** is given in **(e)**. The differential maps **(d,e)** show only small and homogeneous changes of the correlation coefficients, with an

overall RMSD of 0.016 **(d)** and 0.013 **(e)**. The bottom table reports the RMSD for the differential maps calculated between different time windows (i.e., *(i)* last 200 ns – last 400 ns; *(ii)* last 200 ns – last 400 ns) of the native NCP, RAPTA-T–NCP, AUF–NCP, RAPTA-T/AUF–NCP systems and of the RAPTA-T/AUF–NCP system with only one RAPTA-T adduct at site RU1 ("One RAPTA-T").

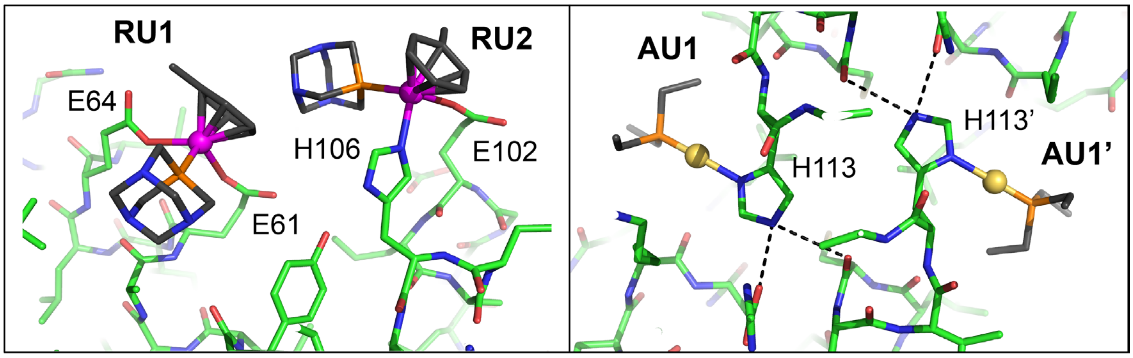

Correlation coefficients (C_{ij}) for the Cα atoms of the residues that bind RAPTA-T and AUF

Site	*i – j (Cα)*	NCP	AUF–NCP	RAPTA-T–NCP	RAPTA-T/AUF–NCP	One RAPTA-T
RU1-RU1	E64 – E61	0.80	0.82	0.86	0.88	0.82
RU2-RU2	H106 – E102	0.78	0.78	0.88	0.86	0.80
RU1-RU2	E64 – H106	0.76	0.72	0.76	0.76	0.81
RU1-RU2	E64 – E102	0.76	0.71	0.82	0.81	0.72
RU1-RU2	E61 – H106	0.68	0.79	0.85	0.82	0.76
RU1-RU2	E61 – E102	0.73	0.75	0.78	0.75	0.76
RU1-AU1	E64 – H113	-0.58	-0.61	0.44	0.61	0.58
RU1-AU1	E61 – H113	-0.69	-0.52	0.45	0.68	0.68
RU1-AU1'	E64 – H113'	-0.72	-0.54	0.36	0.34	0.28
RU1-AU1'	E61 – H113'	-0.61	-0.58	0.38	0.39	0.36
RU2-AU1	H106 – H113	-0.68	-0.62	0.36	0.38	0.30
RU2-AU1	E102 – H113	-0.72	-0.6	0.35	0.42	0.38
RU2-AU1'	H106 – H113'	0.32	0.26	0.38	0.52	0.42
RU2-AU1'	E102 – H113'	0.24	0.21	0.34	0.46	0.46
AU1-AU1'	H113 – H113'	-0.54	-0.46	0.48	0.52	0.47

Correlation coefficients (C_{ij}) for the Ru-Au atoms

i – j (Ru-Au)	RAPTA-T/AUF–NCP	One RAPTA-T
RU1-RU2	0.87	–
RU1-AU1	0.61	0.58
RU1-AU1'	0.38	0.38
RU2-AU1	0.46	–
RU2-AU1'	0.48	–
AU1-AU1'	0.50	0.48

Supplementary Figure 10. Correlation coefficients, extracted from the cross-correlation matrices, for the Cα atoms of the residues that bind RAPTA-T and AUF, reported for all of the simulated systems (large table). For the RAPTA-T/AUF–NCP system, correlation coefficients for the ruthenium and gold atoms are also reported (small table at bottom). The structural image at top provides an overview of the RU1/RU2 and AU1/AU1’ sites.

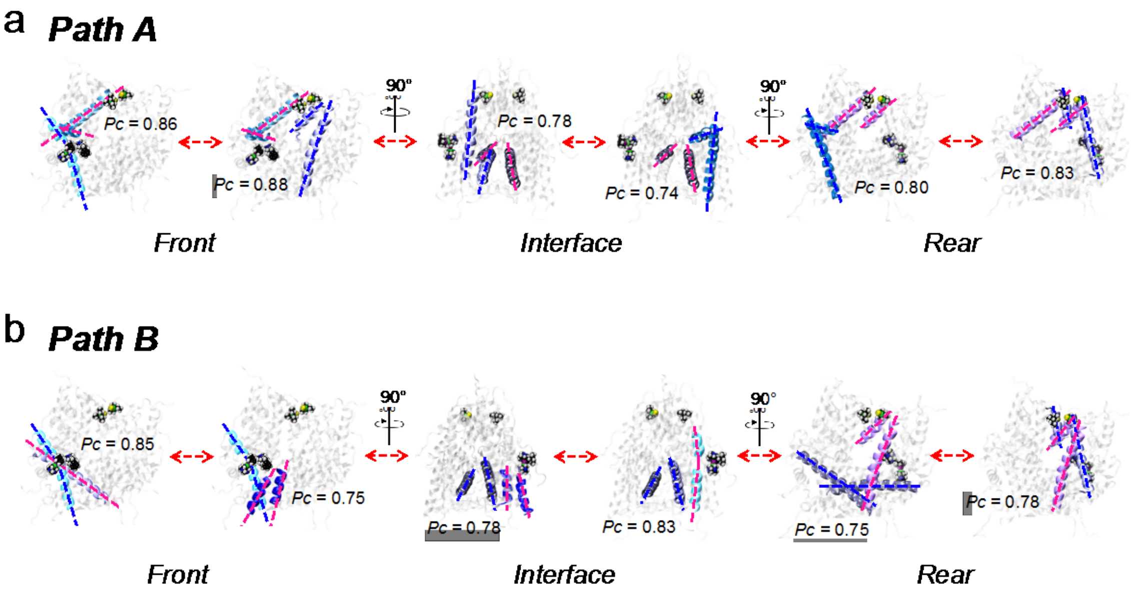

Supplementary Figure 11. Graphical representation of a Pearson correlation coefficient (*Pc*) analysis used to compute the strength of the coupling between the α-helices of the histone components of the RAPTA-T/AUF–NCP system. The highest *Pc* values calculated between pairs of angles formed by the histone α-helices are reported, revealing two correlation paths, *Path A* **(a)** and *Path B* **(b)** that connect the kink of the H2A histone, occurring at the RU sites, with the AUF sites at both front (AU1) and rear (AU1') of the structure. The histone proteins are shown as ribbons. Blue and magenta dashed lines are used to indicate the correlating angles. The structure is rotated of 90° along its main axis, showing the front (left), interface (central panel) and rear (right) sides of the NCP.

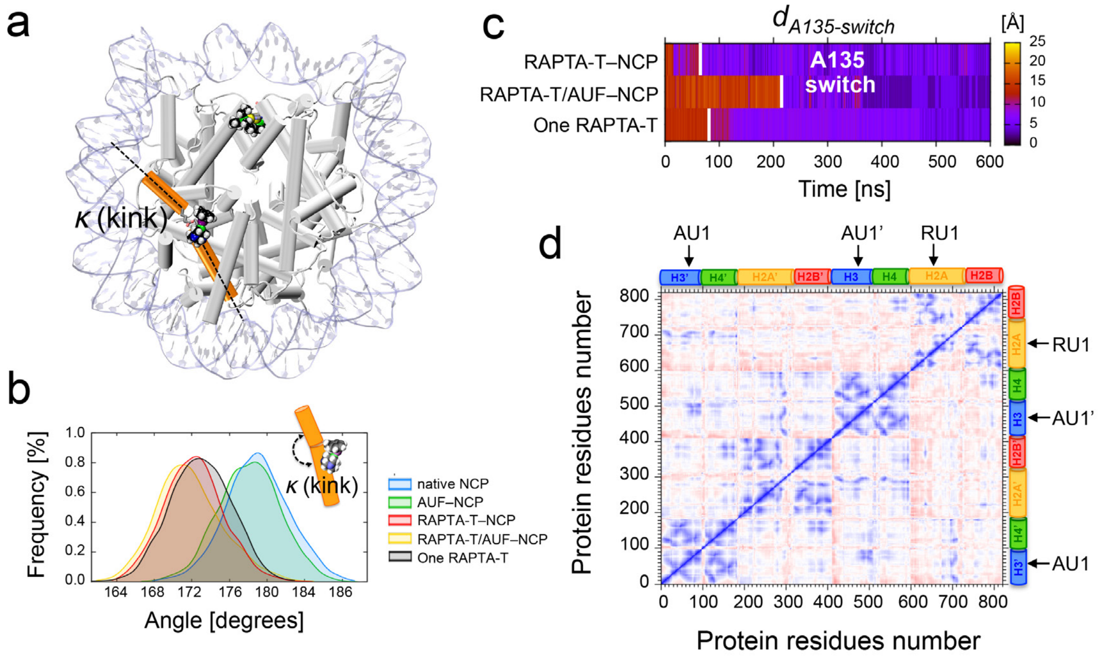

Supplementary Figure 12. Key properties of the RAPTA-T/AUF–NCP system simulated with only a single RAPTA-T adduct at the RU1 site. **(a)** Overview of the simulated system, highlighting the kink angle (κ) within the long α-helix of H2A. **(b)** Probability distribution functions of the kink angle (κ), as calculated during the production runs of the native NCP (blue), AUF–NCP (green), RAPTA-T–NCP (red), RAPTA-T/AUF–NCP (yellow) and RAPTA-T/AUF–NCP having only one RAPTA-T adduct (black) systems. **(c)** Time evolution along MD of the RAPTA-T–NCP, RAPTA-T/AUF–NCP and RAPTA-T/AUF–NCP with only one RAPTA-T adduct systems for the distance ($d_{A135\text{-}switch}$) between the Cα atom of H3 A135 and the center of mass (COM) of the H4 N64 and H4 R67 residues, color-coded according to the scale on the right. **(d)** Cross-correlation matrix of the fluctuations of the Cα atoms (C_{ij}) around their mean positions, calculated over the equilibrium MD trajectory of the RAPTA-T/AUF–NCP with a single RAPTA-T adduct system.

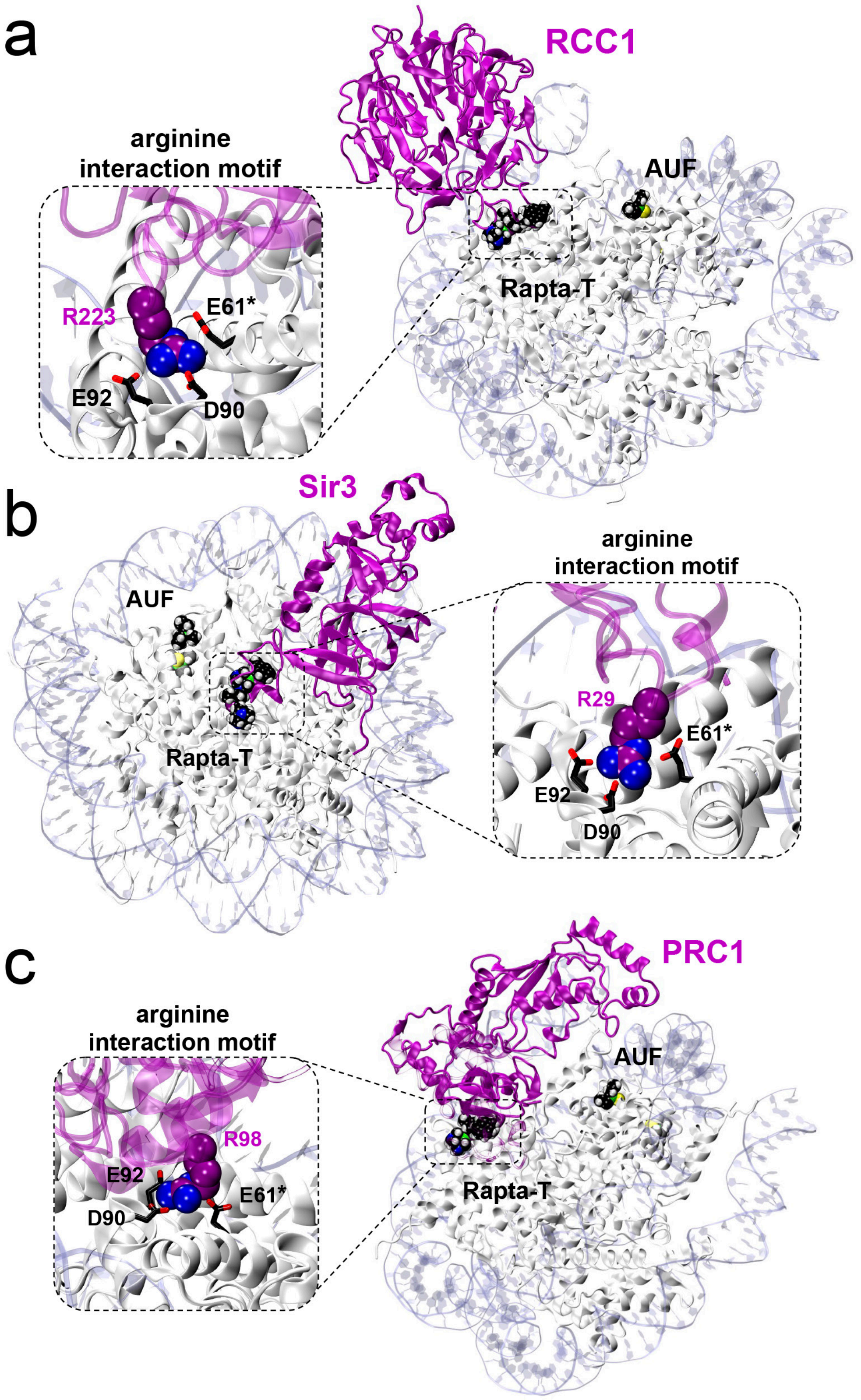

Supplementary Figure 13. **(a-c)** NCP-on-NCP superpositions of the RAPTA-T/AUF–NCP crystal structure with those of either the RCC1[2] (**a**), silent information regulator 3 (Sir3; **b**)[3] or PRC1[4] (**c**) chromatin proteins bound to the NCP (the NCP and

the second chromatin factor molecule associated with the other nucleosome face in the assembly are omitted for clarity). RAPTA-T is seen to overlap with the arginine interaction motif of chromatin factors, suggesting potential for direct (steric) interference with binding of such chromatin factors. For each system, a close-up view of the arginine interaction motif is provided, highlighting the interaction of a key arginine residue with the nucleosome core acidic patch E61, D90, and E92 residues. An asterisk indicates the E61 residue that is engaged in ruthenium ion coordination of RAPTA-T (RU1 site). Chromatin factors (magenta) are shown as ribbons and RAPTA-T, AUF as well as the key arginine residue of chromatin factors are given in space-filling representation.

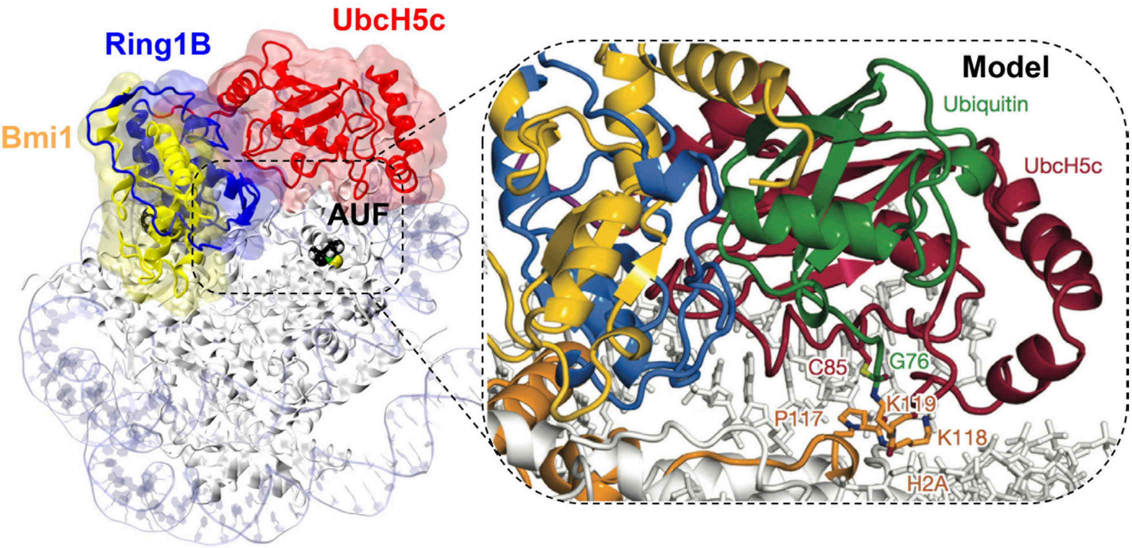

Supplementary Figure 14. NCP-on-NCP superposition of the RAPTA-T/AUF–NCP crystal structure with that of the PRC1 chromatin factor bound to the NCP[4] (the NCP and the second chromatin factor molecule associated with the other nucleosome face in the assembly are omitted for clarity). PRC1 is comprised of the E3 ligase, which is composed of two subunits (Bmi1 and Ring1B, shown in yellow and blue ribbons, respectively) and by the E2 enzyme (UbcH5c, red), which ubiquitylates Lys119 at the H2A C-terminus. AUF is shown in space-filling representation. A close-up view around the AU1 site is given at the right, showing a model for ubiquitin binding in the nucleosome core proposed by McGinty et al[4], which suggests the potential for interference by AUF adducts. Ubiquitin is shown with green ribbons, while the PRC1 ubiquitylation factor is color coded as in the main panel.

SUPPLEMENTARY REFERENCES

1. Collu, F., Vargiu, A., Dreier, J., Cascella, M. & Ruggerone P. Recognition of imipenem and meropenem by the RND-transporter MexB studied by computer simulations. *J. Am. Chem. Soc.* 134, 19146-19158 (2012).
2. Makde R. D., England J. R., Yennawar H. P. & Tan S. Structure of RCC1 chromatin factor bound to the nucleosome core particle. *Nature* **467**, 562-566 (2010).
3. Armache K. J., Garlick J. D., Canzio D., Narlikar G. J., Kingston R. E. Structural basis of silencing: Sir3 BAH domain in complex with a nucleosome at 3.0 Å resolution. *Science* **334**, 977-982 (2011).
4. McGinty, R. K., Henrici, R. C. & Tan, S. Crystal structure of the PRC1 ubiquitylation module bound to the nucleosome *Nature* **514**, 591-596 (2014).
